# Time to castration resistance is a novel prognostic factor of cancer-specific survival in patients with nonmetastatic castration-resistant prostate cancer

**DOI:** 10.1038/s41598-022-20319-z

**Published:** 2022-09-28

**Authors:** Yuji Hakozaki, Yuta Yamada, Taketo Kawai, Masaki Nakamura, Yuta Takeshima, Takuya Iwaki, Taro Teshima, Yoshitaka Kinoshita, Yoichi Fujii, Yoshiyuki Akiyama, Yusuke Sato, Daisuke Yamada, Motofumi Suzuki, Mayu Kashiwagi-Hakozaki, Tetsuo Ushiku, Haruki Kume

**Affiliations:** 1 Department of Urology, The University of Tokyo Graduate School of Medicine, 7-3-1 Hongo, Bunkyo-ku, Tokyo, 163-0033 Japan; 2 Department of Urology, NTT Medical Center, Tokyo, Japan; 3 Department of Urology, The Institute of Medical Science, The University of Tokyo, Tokyo, Japan; 4 Department of Urology, Chiba Tokushukai Hospital, Chiba, Japan; 5 Department of Urology, Tokyo Metropolitan Bokutoh Hospital, Tokyo, Japan; 6 Department of Pathology, Graduate School of Medicine, The University of Tokyo, Tokyo, Japan

**Keywords:** Cancer, Urology

## Abstract

We aimed to identify prognostic factors of cancer-specific survival (CSS) in non-metastatic castration-resistant prostate cancer (M0CRPC) patients. The final analysis of this retrospective cohort included 82 patients who were diagnosed as M0CRPC between 1998 and 2018 at the University of Tokyo Hospital. CRPC was defined as prostate-specific antigen (PSA) progression (increased PSA ≥ 25% and ≥ 2 ng/mL above the nadir or detection of a metastatic lesion). The median value of age and PSA at the time of CRPC were 76 (range 55–94) years and 2.84 (range 2.04–22.5) ng/mL, respectively. The median follow-up time from CRPC diagnosis was 38 (range 3–188) months. The prognostic factors of CSS were ‘PSA doubling time (PSADT) ≤ 3 months’, ‘time to CRPC diagnosis from the start of androgen deprivation therapy (TTCRPC) ≤ 12 months’, of which TTCRPC was a novel risk factor of CSS. In the multivariate analysis, ‘PSADT ≤ 3 months’ and TTCRPC ≤ 12 months’ remained as statistically significant predictors of CSS. Novel risk stratification was developed based on the number of these risk factors. The high-risk group showed a hazard ratio of 4.416 (95% confidence interval 1.701–11.47, C-index = 0.727).

## Introduction

Androgen deprivation therapy (ADT) by orchiectomy or gonadotropin-releasing hormone agonists/antagonists is used as systemic therapy for metastatic prostate cancer and recurrent prostate cancer patients after localized treatment^1^. However, ADT is a palliative treatment, and most patients develop the resistant disease within three years^2^. According to the recommendation of the Prostate Cancer Clinical Trials Working Group 2, castration-resistant prostate cancer (CRPC) is defined as a radiographic progression or prostate-specific antigen (PSA) progression, which is an increase of PSA ≥ 25% and ≥ 2 ng/mL from the nadir, despite testosterone levels of < 50 ng/dL^3^.

Several factors have been identified as predictive markers of metastasis-free and overall survival in M0CRPC patients. Previously reported prognostic markers included baseline PSA^4,5^, PSA velocity^4^, PSA at CRPC diagnosis^6,7^, and PSA doubling time (PSADT)^8,9^. Although CRPC is a lethal disease, prognosis after CRPC diagnosis differs between M0CRPC and M1CRPC. Evidence shows that the median survival with M0CRPC showed ten months longer than M1CRPC^10^. Therefore, risk stratification of survival should be performed separately between M0 and M1CRPC. Among these factors, PSADT is widely used as a predictor of overall survival in M0CRPC patients. Three randomized controlled trials showed survival benefits of darolutamide, enzalutamide, and apalutamide in M0CRPC patients^11–13^. These studies were focused on high-risk patients with shorter PSADT and reported survival benefits in the treatment group. In contrast, the grade 3–4 adverse effect rates were high as 26.3–55.9% in the intervention groups^14^. Taken together, risk assessment is essential for patient selection to maximize therapeutic efficacy and minimize adverse effects.

The objective of this study was to identify prognostic factors for predicting cancer-specific survival (CSS) using clinical data obtained at the time of CRPC diagnosis.

## Results

### Baseline characteristics

The basic characteristics and the inclusion criteria of the 82 patients are shown in Table 1 and Supplementary Fig. 1A. The median value of age, PSA at the time of CRPC was 76 (range 55–94) years and 2.84 (range 2.04–22.5) ng/mL. Pelvic lymph node metastasis was positive in 11 (13.4%) patients. All patients underwent biopsy and 56 (68.3%) had a Gleason score ≥ 8. Regarding localized treatment, 38 (46.3%) patients received radical prostatectomy or radiation therapy. Regarding the type of ADT, most patients received LHRH agonist or surgical orchiectomy (Table 1). Regarding parameters during the ADT treatment, the median value of PSA nadir and PSA reduction rate was 0.18 (range 0.00–14.7) ng/mL and 99.4 (range 71.3–100) %, respectively. The treatment sequence after CRPC diagnosis is shown in Supplementary Fig. 1B. The rates of patients who received docetaxel and ARAT (abiraterone acetate and enzalutamide) as first-line life-prolonging therapy were 28.0% (23/82) and 32.9% (27/82), respectively. Five patients received cabazitaxel as 3rd or 4th line treatment. Thirty-two (39.1%) patients received vintage therapies, including flutamide, estramustine, and low-dose dexamethasone.

**Table 1:** Clinical and histological characteristics of M0CRPC patients.

Patients, n	82
Age at PC diagnosis, years	71 (54–89)
Age at CRPC diagnosis, years	76 (55–94)
PSA at PC diagnosis, ng/mL	25.8 (3.82–534)
PSA at CRPC diagnosis, ng/mL	2.84 (2.04–22.5)
**Clinical stage**	
Tx	12 (14.6)
T1-T2	32 (39.0)
T3	31 (37.8)
T4	7 (8.5)
N0	71 (86.6)
N1	11 (13.4)
**Gleason score**^**a**^**, n**	
6	4 (4.8)
7	22 (26.8)
8	17 (20.7)
≥ 9	39 (47.6)
**Localized treatment**	
None	44 (53.7)
Radical prostatectomy	15 (18.3)
External beam radiation	17 (20.7)
Brachytherapy	6 (7.3)
**Type of ADT**	
Surgical orchiectomy	5 (6.1)
Luteinizing hormone-releasing hormone agonist	76 (92.7)
Luteinizing hormone-releasing hormone antagonist	1 (1.1)
Nadir PSA under ADT treatment, ng/mL	0.18 (0.00–14.7)
PSA reduction rate, %	99.4 (71.3–100)
TnPSA from the start of ADT, months	15 (1–126)
TTCRPC, months	53 (1–190)
PSADT, months	3.6 (0.8–32.4)
**First-line treatment for CRPC**	
Docetaxel	23 (28.0)
Abiraterone acetate	6 (7.3)
Enzalutamide	21 (25.6)
**Other therapies**	
Flutamide	19 (23.2)
Low-dose dexamethasone	9 (11.0)
Estramustine	4 (4.9)
Cycles of docetaxel treatment	6 (2–23)

Continuous variables were reported as the median (range).

*PC* prostate cancer, *PSA* prostate-specific antigen, *ADT* androgen deprivation therapy, *CRPC* castration-resistant prostate cancer, *TnPSA* time to nadir PSA from ADT initiation, *TTCRPC* time to CRPC diagnosis from ADT initiation, *PSADT* PSA doubling time.

^a^At prostate cancer diagnosis.

### Prognostic factors for CSS and MFS

The median follow-up time from CRPC diagnosis was 38 (range 3–188) months. During the follow-up period, 21 patients died of prostate cancer. The 3-year and 5-year CSS rates in the entire cohort were 79.5% and 64.9%, respectively (Supplementary Fig. 1C). ‘Time to CRPC from ADT initiation (TTCRPC)’, and PSADT were identified as prognostic factors of CSS in the univariate analysis (Table 2). ‘TTCRPC ≤ 12 months’ and ‘PSADT ≤ 3 months’ remained as significant factors in the multivariate analysis. Kaplan–Meier curves using the Log-rank tests also showed that ‘TTCRPC ≤ 12 months’ and ‘PSADT ≤ 3 months’ were significant factors of both CSS and MFS (Supplementary Fig. 2A–D).

**Table 2:** Univariate and multivariate analysis of prognostic factors of cancer-specific survival using Cox regression model.

Factors	Univariate analysis		Multivariate analysis	
	Hazard ratio (95% CI)	P value	Hazard ratio (95% CI)	P value
Age at prostate cancer diagnosis, years (≥ 70 vs. < 70)	0.673 (0.273–1.658)	0.3896		
PSA at prostate diagnosis, ng/mL (< 100 vs. ≥ 100)	2.364 (0.695–8.045)	0.1684		
Clinical stage (T3-4 vs. T1-2)	0.766 (0.315–1.860)	0.5556		
TTCRPC, month (≥ 8 vs. < 8)	1.266 (0.509–3.148)	0.6118		
TTCRPC, month (≥ 9 vs. < 9)	1.532 (0.648–3.621)	0.3312		
Local therapy (yes or no)	1.467 (0.604–3.566)	0.5381		
(Radical prostatectomy vs. radiation)	2.144 (0.679–6.767)	0.1936		
Nadir PSA, ng/ml (≥ 0.2 vs. < 0.2)	1.236 (0.510–2.995)	0.6382		
PSA reduction rate, % (< 99.5 vs. ≥ 99.5)	1.636 (0.631–4.241)	0.3107		
TnPSA, months (≤ 12 vs. > 12)	1.768 (0.744–4.204)	0.1969		
Age at CRPC diagnosis, years (≥ 75 vs. < 75)	0.689 (0.289–1.644)	0.4014		
TTCRPC, month (≤ 24 vs. > 24)	2.292 (0.949–5.538)	0.0652		
TTCRPC, month (≤ 12 vs. > 12)	5.692 (1.972–16.42)	0.0013*	3.714 (1.233–11.19)	0.0197*
PSA at CRPC diagnosis, ng/mL (≥ 10 vs. < 10)	0.755 (0.101–5.658)	0.7845		
PSADT, month (≤ 3 vs. > 3)	3.738 (1.496–9.339)	0.0048*	3.005 (1.157–7.809)	0.0239*
**Treatment group**^**a**^				
DOC vs. ARAT	2.323 (0.800–6.757)	0.1212		
Other therapies^b^ alone vs. DOC/ARAT	0.708 (0.259–1.937)	0.5017		

*CI* confidence interval, *PSA* prostate-specific antigen, *TnPSA* time to nadir PSA from ADT initiation, *CRPC* castration-resistant prostate cancer, *TTCRPC* time to CRPC diagnosis from ADT initiation, *ADT* androgen deprivation therapy, *PSADT* PSA doubling time, *DOC* docetaxel, *ARAT* androgen receptor-axis targeted therapies.

*Statistically significant.

^a^All patients were treated by medical/surgical castration ^b^Other therapies include flutamide, estramustine, and low-dose dexamethasone.

**Figure 1:**
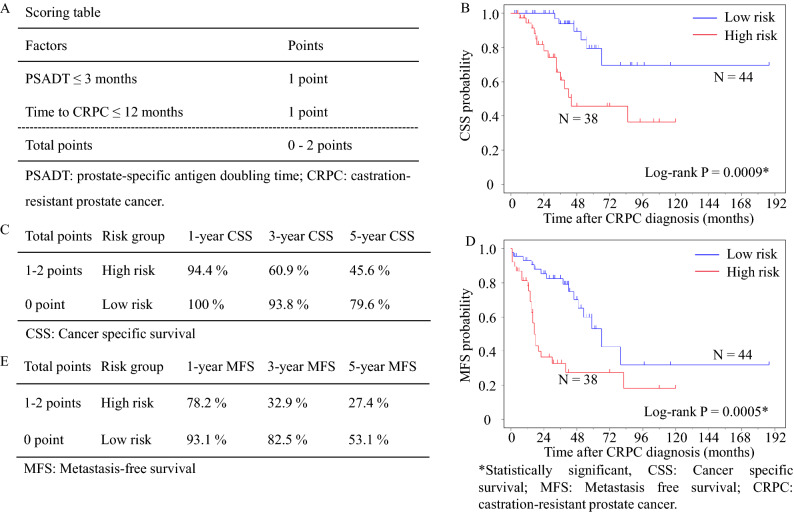
(**A**) Scoring table for risk stratification, (**B**) risk model showing Kaplan–Meier plot of cancer-specific survival, (**C**) cancer-specific survival according to the risk groups, (**D**) risk model showing Kaplan–Meier plot of metastasis-free survival, (**E**) metastasis-free survival according to the risk groups.

Risk stratification was performed based on these factors (Fig. 1A). Figure 1B,C shows the Kaplan–Meier curves of each risk group regarding CSS and MFS, respectively. Specifically, 1-year, 3-year, and 5-year CSS in high and low-risk group were 94.4% vs. 100%, 60.9% vs. 93.8%, and 45.6% vs. 79.6%, respectively (Fig. 1B). Regarding MFS, 1-year, 3-year, and 5-year MFS in high and low-risk group were 78.2% vs. 93.1%, 32.9% vs. 82.5%, and 27.4% vs. 53.1%, respectively (Fig. 1D,E). Obviously, the high-risk group showed a higher risk of cancer-specific deaths and with an HR of 4.416 (95% CI 1.701–11.47, C-index = 0.727) (Supplementary Fig. 3).

**Figure 2:**
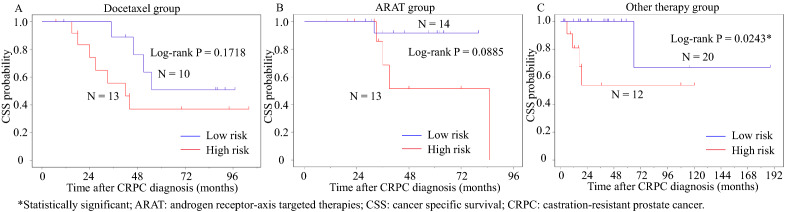
Kaplan–Meier plot of cancer-specific survival of patients treated with (**A**) docetaxel, (**B**) androgen receptor-axis targeted therapies, (**C**) other therapies (vintage therapies) as first-line treatment for CRPC.

Patients were then subdivided into a docetaxel group, an ARAT group, and a vintage therapy group according to the first-line life-prolonging treatment for CRPC (Supplementary Fig. 1B). The Kaplan–Meier curves for the three groups are shown in Fig. 2A–C. Patients with a higher risk had a worse prognosis than any subdivided patients. Notably, a significant difference was observed in other therapy (vintage therapy) group but not in the docetaxel group.

## Discussion

In the present study, we aimed to identify prognostic factors of CSS in M0CRPC patients. Multivariate analysis identified PSADT and TTCRPC as independent predictors of CSS in patients with M0CRPC. TTCRPC was previously reported as a predictor of CSS in CRPC patients^15^. However, this study included both M0 and M1 patients, of which 86% of the 286 patients in the cohort had bone metastases. To our knowledge, this is the first report to identify TTCRPC as a predictor of CSS in M0CRPC patients.

The natural history differs between CRPC patients with and without metastasis. In a study shown by Aly et al., the median survival rates were 13.2 months and 23.2 months in CRPC patients with and without metastasis, respectively^10^. Although CRPC is a lethal disease, nonmetastatic CRPC (M0CRPC) patients show a more favorable prognosis than M1CRPC patients, and risk assessment for M0CRPC patients is essential for designing treatment strategies.

Baseline PSA^4,5^, PSA velocity^4^, PSA at CRPC diagnosis^6,7^, and PSA doubling time (PSADT)^8,9^ are known predictors of survival for M0CRPC patients. Among these factors, a shorter PSADT is a strong predictor of CSS in M0CRPC patients^4,16,17^. This factor is used in the inclusion criteria in the previous randomized controlled studies, which revealed that darolutamide, enzalutamide, and apalutamide were associated with survival benefits in M0CRPC patients with PSADT < 10 months^11–13^. In the present study, ‘PSADT ≤ 3 months’ was also an independent predictor of CSS. Previously, Howard et al. similarly reported that ‘PSADT < 3 months’ is a predictor of CSS^8^. However, PSADT was calculated according to PSA levels after the diagnosis of CRPC. In the present study, PSADT was calculated using PSA levels before CRPC diagnosis, which may hold clinical significance in terms of decision-making of initial-treatment options.

We subdivided patients according to the first-line treatment regimen and found that this risk stratification was well fit especially in ARAT and vintage groups. TTCRPC is reported as a predictor of sensitivity to ARAT drugs in mCRPC patients^18^ and this might relate to the difference of CSS in the ARAT group. While the analysis in the docetaxel group is limited by its small sample size and showed no significant difference, patients with the high-risk group showed poor survival.

Prostate-specific membrane antigen positron emission tomography (PSMA-PET) is a new rising method that can evaluate metastatic lesions with high specificity. PSMA-PET detected pelvic and M1 disease in 44% and 55% of the patients with negative conventional imaging, respectively, including 98 patients with Gleason score of ≥ 8 or PSADT ≤ 10 months^19^. In the present study, a shorter TTCRPC was associated with higher hazard ratios, suggesting that M0CRPC patients with early relapse after ADT initiation are potential candidates for receiving this imaging technique.

This study had several limitations. First, there may be a selection bias regarding treatment options since the choice of therapy may have depended on the patient’s severity of the disease. Although we divided patients into subgroups according to the first-line life-prolonging treatment, we cannot entirely ignore this bias. Second, this is the retrospective cohort in the single university hospital. There may be an unintentional selection bias in the recruitment process of the patients. Third, new imaging techniques such as DWIBS (Diffusion-weighted Whole body Imaging with Background Suppression) or PSMA-PET (Prostate-Specific Membrane Antigen -ligand Positron Emission Tomography) have been introduced during the period of enrollment in the present study. Therefore, we may have had cases involved with hidden metastasis that may have been detected by these new techniques. Forth, although Docetaxel and Abiraterone were not clinically used for non-metastatic CRPC in general worldwide, the use of these agents has been available for CRPC patients with/without metastasis in Japan. And finally, one of the major limitations of this study include the small sample size of the cohort. A prospective study with a larger sized population may be required to externally validate and confirm our model.

In summary, we identified a shorter PSADT and TTCRPC as independent prognostic factors of CSS in M0CRPC patients. These patients may be candidates for novel imaging techniques such as PSMA-PET, and early treatment may be beneficial to prolong the survival of these patients.

## Methods

### Study design and patient selection

This retrospective cohort included 82 consecutive patients with nonmetastatic CRPC that were diagnosed between 1998 and 2018 at the University of Tokyo Hospital. CRPC was defined as PSA progression or radiological progressions under castrated. Castration was defined as testosterone < 50 ng/dL or ADT by either using surgical orchiectomy or luteinizing hormone-releasing (LHRH) agonist or antagonist^8^. PSA and radiographic progression were described as an increase in PSA of ≥ 25% and ≥ 2 ng/mL above the nadir and the appearance of ≥ 2 lesions according to the Prostate Working Group 2 (PCWG2) recommendation^20^. Notably, the clinical use of Docetaxel, Abiraterone, and Enzalutamide has been available in nonmetastatic CRPC patients in Japan from when these agents were first introduced. The general dose of Docetaxel was 75 mg/m^2^ every 3 weeks. A total of 24 patients were excluded because of incomplete information, including the nadir PSA level or treatment at CRPC diagnosis (Supplementary Fig. 1A). Additionally, 2 patients were excluded due to poor systemic conditions to receive pharmaceutical drugs or concomitant aggressive malignancies and inability to evaluate metastatic lesions. Finally, 11 patients with unproven diagnosis by prostate biopsy were excluded (Supplementary Fig. 1A). To note, the presence of metastasis was confirmed by imaging tests, including bone scans and computed tomography of the chest, abdomen, and pelvis. M0CRPC was defined as the absence of metastatic lesions by the last imaging test before CRPC diagnosis^8^. In total, 82 M0CRPC patients met the criteria. The protocol for this research project was approved by the ‘Ethics Committee of the Tokyo University Hospital’ and performed according to the provisions of the Declaration of Helsinki (# 3124). Regarding the present study, ‘Ethics Committee of the Tokyo University Hospital’ waived the requirement of the written informed consent.

### Data retrieval

Clinical parameters including age, Gleason score before treatment, clinical TNM stage and PSA level at prostate cancer diagnosis, presence of local therapy, the nadir PSA level from the start of ADT before CRPC diagnosis, CRPC treatments were retrospectively reviewed from the electrical medical records system of the University of Tokyo Hospital. CSS was defined as ‘the time from CRPC diagnosis to death from prostate cancer’. MFS was defined as ‘the time from CRPC diagnosis to radiological evidence of metastasis’. Gleason score was determined based on the grading system reported in 1977^21^ and revised criteria^22^ at the time of biopsy. TTCRPC was defined as the duration between the CRPC diagnosis and ADT initiation. PSADT was calculated using a web-based calculator from Memorial Sloan Kettering Cancer Center (https://www.mskcc.org/nomograms/prostate/psa_doubling_time). All PSA levels within six months before CRPC diagnosis were used for the PSADT calculation. PSA levels before achieving nadir PSA or < 0.2 ng/mL were excluded from the calculation^23^. The rate of PSA reduction was calculated using the following formula: 1 − (nadir PSA)/(initial PSA)^24^. Time to nadir PSA (TnPSA) was the time from the start of ADT to the date on which the patient achieved the lowest PSA value for the first time during ADT treatment and before CRPC diagnosis^25^.

### Statistical analysis

Univariate and multivariate analyses were performed using the Cox proportional hazards regression model to identify independent factors predicting CSS duration. Median values were used to calculate the cut-off for continuous variables in the analysis. The cut-off value of PSA was rounded off for clinical usage. The cut-off values were determined for TTCRPC^26^, PSADT^8^, nadir PSA^27^, TnPSA^28^ and CRPC diagnosis^4^ as described previously. Statistically significant prognostic factors in univariate analysis were included in multivariate analysis. Independent prognostic factors were included in multivariate analysis. Risk stratification was performed according to the number of these risk factors. CSS and MFS were determined in each risk group using the Kaplan–Meier method. The two-sided log-rank test was performed to evaluate the difference of CSS and MFS among risk groups. The hazard ratio and concordance index (C-index) were calculated using the Cox proportional hazards regression model. Patients were divided into three groups as follows: those who received docetaxel as first-line life-prolonging therapy, patients treated with androgen receptor-axis targeted therapies (ARAT), and those who never received docetaxel nor ARAT but had other therapies (vintage therapies), including flutamide, estramustine, and low-dose dexamethasone. Life-prolonging therapy was defined as the drugs which showed an increase in overall survival compared with the control group based on the randomized controlled trials such as docetaxel^29^, abiraterone^30^, enzalutamide^31^, and cabazitaxel^32^. CSS and MFS were determined using the Kaplan–Meier method according to the number of prognostic factors. Statistical analyses were performed using JMP 16.1.0 (SAS Institute Inc., Cary, NC, USA), and C-index was calculated by R Version 3.6.0 (Comprehensive R Archive Network), and a p value < 0.05 was considered statistically significant.

## Supplementary Information

Supplementary Information.

## Data Availability

The dataset used in the present study is not publicly available since there are ongoing clinical studies based on this same dataset, but it can be used by a reasonable request to the corresponding author.

## References

[1] Uchio EM, Aslan M, Wells CK, Calderone J, Concato J (2010). Impact of biochemical recurrence in prostate cancer among us veterans. Arch. Int. Med..

[2] Chandrasekar T, Yang JC, Gao AC, Evans CP (2015). Mechanisms of resistance in castration-resistant prostate cancer (CRPC). Transl. Androl. Urol..

[3] Luo J, Beer TM, Graff JN (2016). Treatment of nonmetastatic castration-resistant prostate cancer. Oncology (Williston Park).

[4] Smith MR (2005). Natural history of rising serum prostate-specific antigen in men with castrate nonmetastatic prostate cancer. J. Clin. Oncol..

[5] Smith MR, Cook R, Lee KA, Nelson JB (2011). Disease and host characteristics as predictors of time to first bone metastasis and death in men with progressive castration-resistant nonmetastatic prostate cancer. Cancer.

[6] Moreira DM (2016). Predictors of time to metastasis in castration-resistant prostate cancer. Urology.

[7] Miyake H (2020). Assessment of factors predicting disease progression in Japanese patients with non-metastatic castration-resistant prostate cancer. Anticancer Res..

[8] Howard LE (2017). Thresholds for PSA doubling time in men with non-metastatic castration-resistant prostate cancer. BJU Int..

[9] Smith MR (2013). Denosumab and bone metastasis-free survival in men with nonmetastatic castration-resistant prostate cancer: Exploratory analyses by baseline prostate-specific antigen doubling time. J. Clin. Oncol..

[10] Aly M (2020). Survival in patients diagnosed with castration-resistant prostate cancer: A population-based observational study in Sweden. Scand. J. Urol..

[11] Fizazi K (2020). Nonmetastatic, castration-resistant prostate cancer and survival with darolutamide. N. Engl. J. Med..

[12] Sternberg CN (2020). Enzalutamide and survival in nonmetastatic, castration-resistant prostate cancer. N. Engl. J. Med..

[13] Smith MR (2021). Apalutamide and overall survival in prostate cancer. Eur. Urol..

[14] Chung DY, Ha JS, Cho KS (2021). Novel treatment strategy using second-generation androgen receptor inhibitors for non-metastatic castration-resistant prostate cancer. Biomedicines.

[15] Bournakis E (2011). Time to castration resistance is an independent predictor of castration-resistant prostate cancer survival. Anticancer Res..

[16] Whitney CA (2019). Impact of age, comorbidity, and PSA doubling time on long-term competing risks for mortality among men with non-metastatic castration-resistant prostate cancer. Prostate Cancer Prostatic Dis..

[17] Freedland SJ (2007). Death in patients with recurrent prostate cancer after radical prostatectomy: Prostate-specific antigen doubling time subgroups and their associated contributions to all-cause mortality. J. Clin. Oncol..

[18] Loriot Y (2015). Prior long response to androgen deprivation predicts response to next-generation androgen receptor axis targeted drugs in castration resistant prostate cancer. Eur. J. Cancer.

[19] Fendler WP (2019). Prostate-specific membrane antigen ligand positron emission tomography in men with nonmetastatic castration-resistant prostate cancer. Clin. Cancer Res..

[20] Scher HI (2008). Design and end points of clinical trials for patients with progressive prostate cancer and castrate levels of testosterone: Recommendations of the prostate cancer clinical trials working group. J. Clin. Oncol..

[21] Gleason DF, Tannenbaum M (1977). Histologic grading and clinical staging of prostatic carcinoma. Urologic Pathology: The Prostate Philadelphia.

[22] Epstein JI, Allsbrook WC, Amin MB, Egevad LL, Committee IG (2005). The 2005 international society of urological pathology (ISUP) consensus conference on Gleason grading of prostatic carcinoma. Am. J. Surg. Pathol..

[23] Arlen PM (2008). Prostate specific antigen working group guidelines on prostate specific antigen doubling time. J. Urol..

[24] Sasaki T, Onishi T, Hoshina A (2012). Cutoff value of time to prostate-specific antigen nadir is inversely correlated with disease progression in advanced prostate cancer. Endocr. Relat. Cancer.

[25] Choueiri TK (2009). Time to prostate-specific antigen nadir independently predicts overall survival in patients who have metastatic hormone-sensitive prostate cancer treated with androgen-deprivation therapy. Cancer.

[26] Chi K (2015). Treatment of mCRPC in the AR-axis-targeted therapy-resistant state. Ann. Oncol..

[27] Kim M (2015). Prostate-specific antigen kinetic profiles during androgen deprivation therapy as prognostic factors in castration-resistant prostate cancer. Urol. Oncol..

[28] Fukuoka K (2020). Predictors of poor response to first-generation anti-androgens as criteria for alternate treatments for patients with non-metastatic castration-resistant prostate cancer. Int. Urol. Nephrol..

[29] Tannock IF (2004). Docetaxel plus prednisone or mitoxantrone plus prednisone for advanced prostate cancer. N. Engl. J. Med..

[30] Fizazi K (2012). Abiraterone acetate for treatment of metastatic castration-resistant prostate cancer: Final overall survival analysis of the COU-AA-301 randomised, double-blind, placebo-controlled phase 3 study. Lancet Oncol..

[31] Beer TM (2014). Enzalutamide in metastatic prostate cancer before chemotherapy. N. Engl. J. Med..

[32] de Bono JS (2010). Prednisone plus cabazitaxel or mitoxantrone for metastatic castration-resistant prostate cancer progressing after docetaxel treatment: A randomised open-label trial. Lancet.

