# Supplementary material for: Time to castration resistance is a novel prognostic factor of cancer-specific survival in patients with nonmetastatic castration-resistant prostate cancer
Source: Sci Rep. 2022 Sep 28;12:16202. doi: 10.1038/s41598-022-20319-z (PMC9519913; doi:10.1038/s41598-022-20319-z)
Supplement: Supplementary file 1 — Supplementary Information. [file 41598_2022_20319_MOESM1_ESM.pdf]

Time to castration resistance is a novel prognostic factor of cancer-specific survival in patients with nonmetastatic castration-resistant prostate cancer

Yuji Hakozaki, Yuta Yamada*, Taketo Kawai, Masaki Nakamura, Yuta Takeshima, Takuya Iwaki, Taro Teshima, Yoshitaka Kinoshita, Yoichi Fujii, Yoshiyuki Akiyama, Yusuke Sato, Daisuke Yamada, Motofumi Suzuki, Mayu Kashiwagi-Hakozaki, Tetsuo Ushiku, Haruki Kume

Supplementary Fig. 1

A Flowchart of inclusion and exclusion criteria

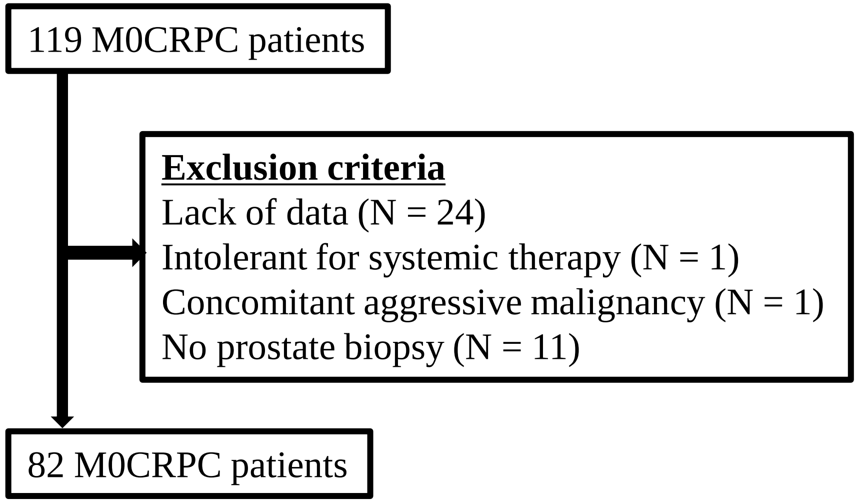

M0CRPC: nonmetastatic castration-resistant prostate cancer.

C Kaplan-Meier plot of cancer-specific survival

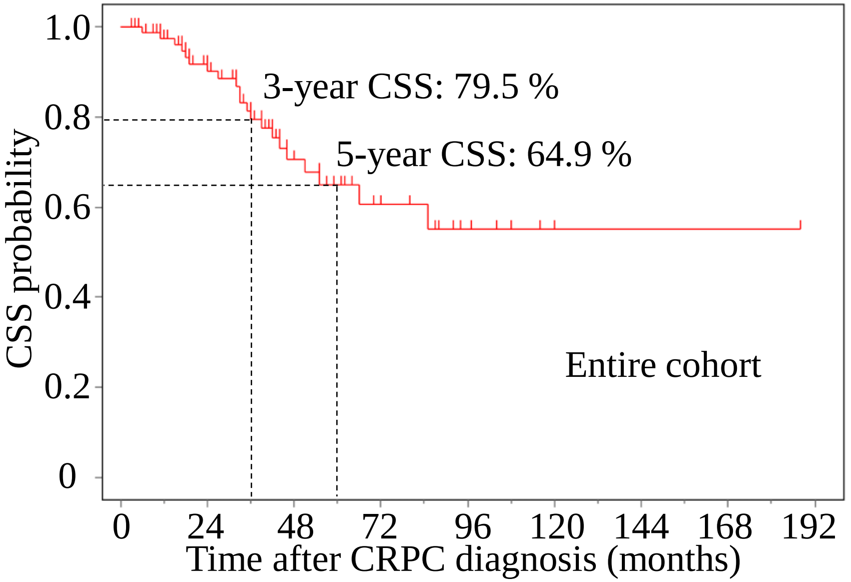

CSS: Cancer-specific survival.

B Treatment sequences for M0CRPC

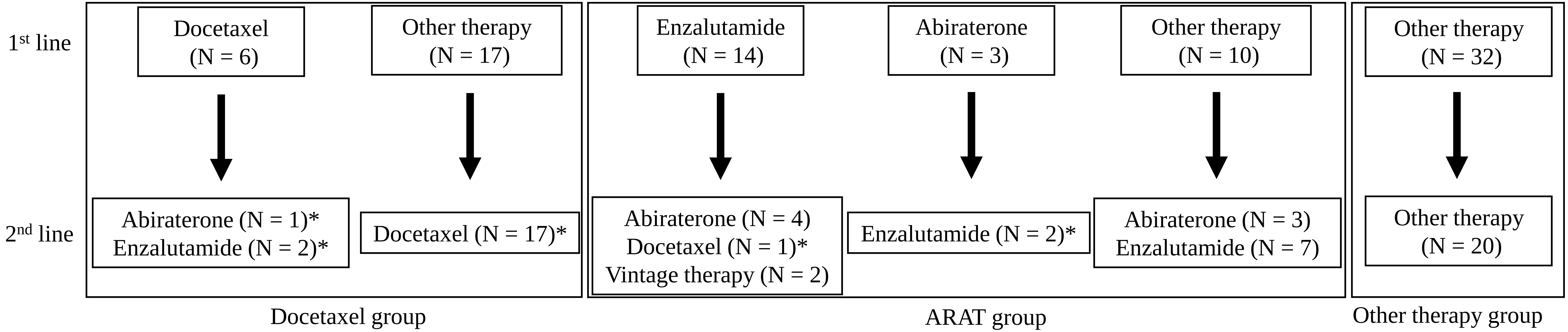

*Five patients received cabazitaxel as 3rd or 4th line therapy. ARAT: androgen receptor-axis targeted therapies.

Supplementary Fig. 2 Kaplan-Meier plot of cancer-specific and metastatic free survival of prognostic factors

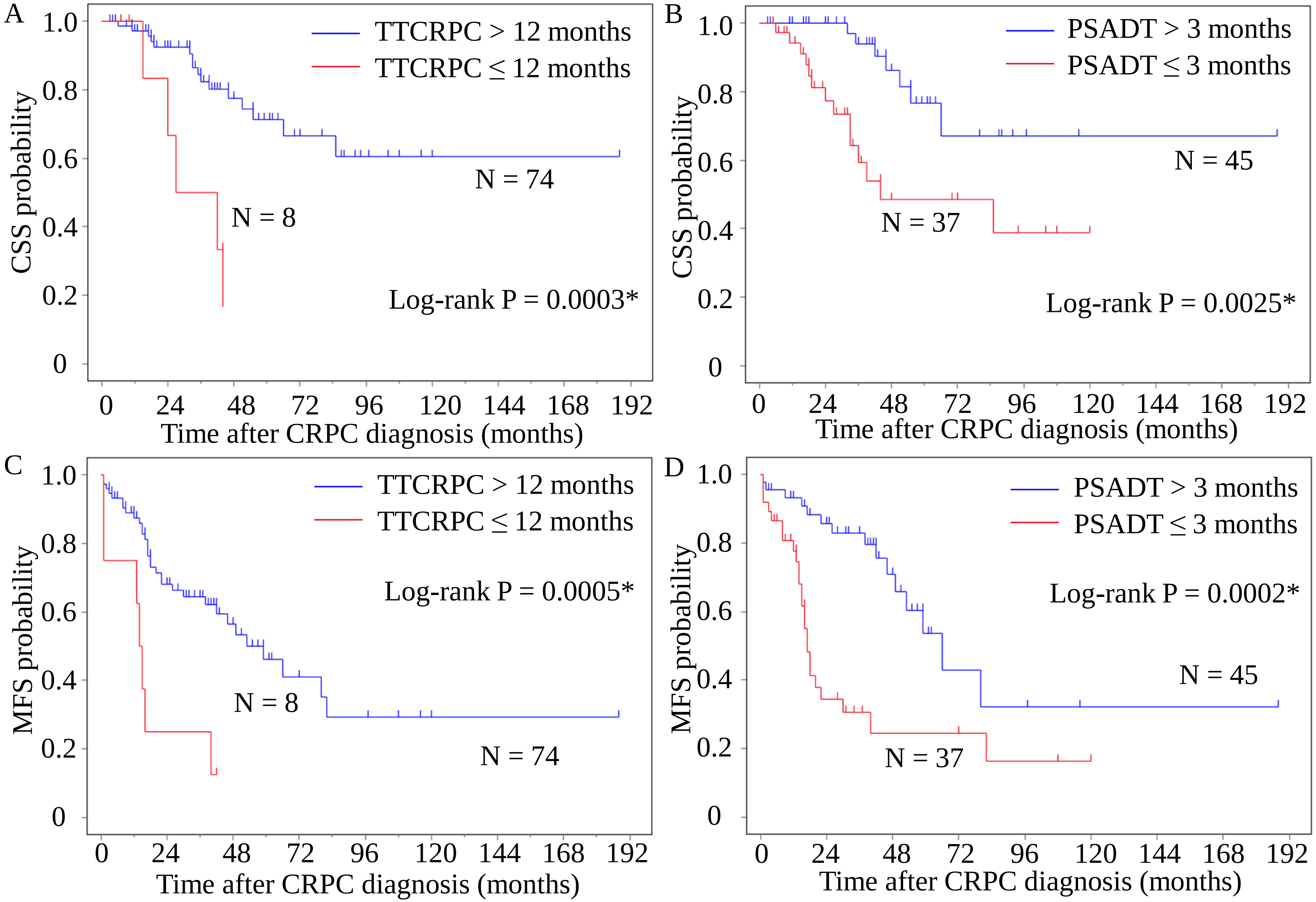

*Statistically significant, CSS: Cancer specific survival; MFS: Metastasis free survival; CRPC: castration-resistant prostate cancer; TTCRPC: Time to CRPC; PSADT: Prostate-specific antigen doubling time.

Supplementary Fig. 3 Hazard ratios and C-index of cancer-specific survival according to the risk group and identified predictive factors

	Hazard ratio	C-index	P-value
Present risk model (High risk vs. Low risk)	4.416 (1.701-11.47)	0.727	0.0023*
PSADT (≤ 3 mo. vs. > 3 mo.) alone	3.738 (1.496-9.339)	0.712	0.0048*
TTCRPC (≤ 12 mo. vs. > 12 mo.) alone	5.692 (1.972-16.42)	0.597	0.0013*

*Statistically significant, mo: months; PSADT: Prostate-specific antigen doubling time; TTCRPC: Time to castration-resistant prostate cancer
